# From game engagement to craving responses – The role of gratification and compensation experiences during video-gaming in casual and at-risk gamers

**DOI:** 10.1016/j.abrep.2023.100520

**Published:** 2023-11-30

**Authors:** S. Antons, M. Liebherr, M. Brand, A. Brandtner

**Affiliations:** a General Psychology: Cognition and Center for Behavioral Addiction Research (CeBAR), University of Duisburg, Duisburg, Germany; b Erwin L. Hahn Institute for Magnetic Resonance Imaging, Essen, Germany

**Keywords:** Gaming, Craving, Cue-reactivity, Flow, Engagement, Immersion

## Abstract

**Introduction:**

Although playing videogames is a common leisure activity some individuals develop problematic gaming behaviors or even symptoms of a gaming disorder. Game engagement may be involved in reinforcement learning that may result in experiences of craving, an important feature of gaming disorder. In the following study we aimed to approach the question which aspects contribute to increased craving for gaming.

**Methods:**

Overall, 439 individuals participated in an online survey, answering questionnaires on game engagement, experience of gratification and compensation, craving, and symptoms of gaming disorder. A mediation model testing if the association between game engagement and facets of craving are mediated by the experience of gratification and compensation during gaming.

**Results:**

Three facets of craving (reward/relief, physiological, obsessive craving) were statistically explained by game engagement and the experience of gratification and compensation. Models differed between casual gamers and at-risk gamers. The effects on reward/relief and physiological craving were fully mediated in the casual group and partially mediated in the at-risk group by gratification and compensation experiences.

**Conclusion:**

Game engagement may contribute to the development of craving by increasing the experience of gratification and compensation and thus might be important in reinforcement learning. Games evoking a high engagement may therefore have a higher addictive potential. Further processes potentially related to game engagement, such as intrusive thoughts and desire thinking, which are related to craving experiences, should be considered in future studies.

## Highlights

• Facets of craving are related to game engagement (e.g., flow, immersion)
• The experience of gratification and compensation mediate these effects in gamers.
• Differences between casual and at-risk gamers indicate increasing habitualization.
• Game engagement is not per se problematic but increase addictive potential of games.
• Author Credit Statement.
• **Stephanie Antons:** Conceptualization Methodology Investigation Formal analysis Writing - Original Draft.

## 1 Introduction

Playing videogames is a common leisure activity associated with many positive effects if done in a functional and enriching manner (Jones et al., 2014, Raith et al., 2021, Villani et al., 2018). For example, previous studies identified a gaming-related increase in positive emotions and reduction of negative emotions. Gaming has been associated with an improvement of relationships experiences (especially to other players), the increase of engagement in personally meaningful activities, and the creation of feelings of competence or mastery. Furthermore, games may provide the opportunity to escape from real life within a virtual world. These positive experiences have been associated with increased mental health and well-being (Jones et al., 2014, Raith et al., 2021, Villani et al., 2018). However, when individuals engage excessively in videogames, the positive effects sometimes turn into negative ones, affecting important areas of daily life and/or causing marked distress (Altintas et al., 2019, Mihara and Higuchi, 2017, Montag and Pontes, 2023, Wartberg et al., 2019). If the excessive use manifests over a longer period of time and individuals feel unable to control or stop their gaming without external influence or intervention, gaming may become pathological (King & Potenza, 2019). The pathological and addictive use of videogames is classified as gaming disorder (GD) under the category of disorders due to addictive behaviors in the International Classification of Diseases, 11th Revision (ICD-11). Gaming disorder is characterized by (1) impaired control over gaming, (2) increasing priority given to gaming to the extent that the behavior takes precedence over other interests and everyday activities, and (3) continuation or escalation of the gaming behavior despite the occurrence of negative consequences (World Health Organization, 2022). Prevalence estimates depict that about 2.38 to 3.91 % of people worldwide may suffer from dysregulated and addictive gaming (Stevens et al., 2021), indicative of the relevance of GD as a mental health problem.

Craving for gaming describes the intense and urgent desire to play videogames (Antons et al., 2020). Based on theories (Brand et al., 2019, Wei et al., 2017) and current evidence (Antons et al., 2020, Brandtner et al., 2020, Yen et al., 2022, Zhou et al., 2021) it is assumed that craving is a central mechanism in the development and maintenance of GD. Here, craving has not only been associated with increased symptom severity (Antons et al., 2020, Brandtner et al., 2020, Diers et al., 2023, Yen et al., 2022, Zhou et al., 2021) but also with shorter abstinence (Yen et al., 2022). Craving may in itself be subdivided into sub-facets that differ with regard to experiential properties (May et al., 2014). Its qualities might vary over time where initial rewarding craving content might transit into more obsessive craving experiences throughout the development of addictive (obsessive) behavior patterns (Brand, 2022). Skinner and Aubin (2010) identified a range of craving models that either pronounce conditioning processes, psychobiological and motivational factors, or cognitive information-processing mechanisms. As an approach to extract the most dominant convergence among these models, Flaudias et al. (2019) propose a metacognitive hub model of craving that makes a general distinction between 1) cognitive/obsessive craving, 2) automatic craving, and 3) physiological craving; where each component may be experienced more or less explicitly.

Although there is strong evidence for involvement of craving in addictive behaviors including gaming (Starcke et al., 2018, Tiffany and Wray, 2012), factors that contribute to the development of craving for gaming have been less in focus of empirical research. When considering the origin of craving from a theoretical perspective, it is assumed that craving is the result of associative learning (classical and operant conditioning; Brand et al., 2019, Tiffany and Wray, 2012). Due to these conditioning processes, previously unrelated stimuli may become triggers that induce a craving for engaging in a behavior (e.g., gaming). The conditioning process can be so far-reaching that this craving for a secondary reward (i.e., gaming) is higher than the craving elicited by cues of primary rewards such as food (Zhou et al., 2021). The key of conditioning processes is that the probability of a behavior execution increases if the behavior is associated with a gratifying (rewarding) or compensating (relieving) consequence (Staddon & Cerutti, 2003). Accordingly, and as proposed in the Interaction of Person-Affect-Cognition-Execution Model (I-PACE Model; Brand et al., 2019), craving for gaming should be strongly associated to specific features of games that elicit the experiences of gratification and compensation.

One main characteristic of videogames is the potential to deeply engage users. Constructs that have been investigated in this context are immersion, presence, flow, psychological absorption, and dissociation (Brockmyer et al., 2009). To get deeply immersed into a videogame has been identified as one central motive to play games especially for individuals with high symptom severity of GD (Wang & Cheng, 2022). This engagement can lead to positive and gratifying experiences. In addition, high game engagement may lead to a high relief from psychological stress by dissociating from oneself and one’s problems (Casale et al., 2021, Guglielmucci et al., 2019). These actual experiences during gaming need to be distinguished from gaming using motives that may drive gaming behavior but are more associated with the expected outcome of the behavior (Wegmann et al., 2022). Actual gaming experiences such as facets of game engagement (experience of presence and flow) have been associated with increased symptoms of GD (Chou and Ting, 2003, Hu et al., 2019, Li et al., 2021, Stavropoulos et al., 2019). Thus, due to the engagement into the game, individuals may experience a certain gratification and compensation while playing videogames.

Until recently, the concrete experiences of gratification and compensation, beyond general using motives, have not been in the focus of research on GD. With the development of two questionnaires measuring the experience of gratification and the experience of compensation in various behaviors including gaming, Wegmann et al. (2022) enable the systematic investigation of these reinforcing factors in the context of GD. Based on the factor structure it can be assumed that an experience of gratification may be considered by feelings of pleasure (feeling good, experiencing fun, feeling satisfied) or the satisfaction of needs (closeness to others, feeling successful, feeling powerful). Similarly, an experience of compensation may occur through the compensation of unsatisfied needs (feeling less unsuccessful, powerless, or lonely) and the relief from negative feelings (feeling less constricted, insecure, or worried). All four factors were positively associated with symptom severity of GD and increased craving for gaming (Wegmann et al., 2022). In line with these results, previous studies showed that individuals with higher symptoms of addictive use of videogames show a decreased general satisfaction of needs, and higher motivation to play videogames for escapism or to increase interpersonal relationships (Przybylski et al., 2009, Wang and Cheng, 2022). Thus, features of videogames may satisfy or compensate for these needs. On the other hand, the study by Przybylski et al. (2009) showed that individuals with obsessive gaming passion show reduced enjoyment during gaming. Thus, it can be assumed that the experience of pleasure might be less relevant in the maintenance of GD compared to the satisfaction of needs and features of compensation. While gratifying or compensating processes take place, both may act as reinforcing factors that could contribute to the development of craving.

Based on this theoretical and empirical background, the current work aims to contribute to the question which factors (statistically) explain craving experiences. In particular, we investigate in which way game engagement is associated with increased intensity of craving for gaming and how this association is mediated by the experience of gratification and compensation. Since craving is a multidimensional construct (Flaudias et al., 2019), we will differentiate between three different facets of craving: reward/relief craving, physiological craving, and obsessive craving. By doing so, we will separately explore the different contribution of reinforcement mechanisms (i.e., gratification and compensation) in casual gaming behaviors and at-risk stages of problematic gaming.

## 2 Methods

### 2.1 Participants

Overall, 439 individuals who indicated to having played videogames in the foregone four weeks (*M*_age_ = 34.31, *SD* = 8.92, range: 18–49 years; 47.4 %/52.6 %/0% female/male/non-binary; video-gaming in h/week *M* = 12.87, *SD* = 19.57), were examined. Participants were recruited through an online panel-provider. Participants received a monetary incentive from the panel provider of 2.30 Euro in return of the 0.5 h lasting study. The study protocol was approved by the local ethics committee.

### 2.2 Measures

#### 2.2.1 Game engagement

The Game Engagement Questionnaire (GEQ; Brockmyer et al., 2009) was used to measure the extent to which individuals experience absorption, flow, presence, and immersion into the game. These factors constitute the subscales of the questionnaire that contains 19 items (e.g., “Time seems to kind of stand still or stop”) answered on a five-point Likert scale ranging from 1 = no to 3 = more or less to 5 = yes. For each subscale (i.e., absorption, flow, presence, and immersion) and for the whole scale, mean scores are calculated, where higher scores indicate higher game engagement. Cronbach’s alpha/ McDonalds’ Omega were 0.870/0.869 for the whole scale, 0.708/0.743 for the subscale absorption, 0.743/0.735 for the subscale flow, and 0.556/0.545 for the subscale presence (only one item for the subscale immersion). Given that we planned to model game engagement on latent level, the factor loadings will demonstrate if the overall latent dimension will be adequately represented by the four factors, even though the internal consistency of one factor is low.

#### 2.2.2 Experience of gratification/experience of compensation

The German Experience of Gratification Scale (EGS) and Experience of Compensation Scale (ECS; Wegmann et al., 2022) were used to measure experienced reinforcement while playing games. The EGS, consisting of two sub-scales, measures with 3 items each the extent that individuals encounter a gratification of needs and experience of pleasure (e.g., “While gaming I feel good”) while the ECS measures a compensation of needs and experience of relief with 3 items for each sub-scale (e.g., “While gaming I feel less lonely”). All items are rated on a five-point Likert scale ranging from 0 = never to 4 = very often. Mean scores are calculated with a potential range of 0–4 and higher scores indicating a greater experience of gratification or compensation. Cronbach’s alpha and McDonalds’ omega were 0.692/0.698 for the EGS gratification of needs, 0.568/0.578 for the EGS experience of pleasure, 0.849/0.854 for the EGC compensation of needs and 0.851/0.854 for the ECS experience of relief from negative feelings. Internal consistency of the experience of pleasure scale is at the edge of acceptable. The reliability (Cronbach’s alpha/ McDonalds’ omega) for the whole EGS and ECS scales showed good reliability with 0.748/0.756 and 0.911/0.911, respectively.

#### 2.2.3 Craving

The experience of craving was measured with the German Craving Assessment Scale for Behavioral Addictions (CASBA; Antons et al., 2019). The scale measures craving with 9 items which were developed based on the craving components proposed by Verheul et al. (1999). Thus, items reflect reward and relief craving (e.g., “Playing computer games now would be best to improve my mood.”), obsessive craving (e.g., “Playing computer games now is the most urgent thing I want do to”), and physiological craving (e.g., “Playing computer games now would cause an intense sense of well-being in me”). Each item is answered on a five-point Likert scale with 1 = completely disagree to 5 = completely agree. A sum score is calculated resulting in a possible range of 5–45 with higher scores being indicative of higher craving. In this sample, the CASBA showed a good reliability with Cronbach’s alpha/ McDonalds’ omega of 0.910/0.909.

#### 2.2.4 Symptoms of addictive videogaming

Symptom severity, as measured with a German translation of the Internet Gaming Disorder Test (IGDT-10; Király et al., 2017), was used to discriminate between casual (*n* = 255) and at-risk (*n* = 184) videogaming according to provided cut-off scores. So far, there is no German validation of the IGDT-10 wherefore we used a German translation that was previously used on a German sample (Brandtner et al., 2020). This self-report measurement is constructed on the basis of the DSM-5 criteria of gaming disorder (American-Psychiatric-Association, 2013). Each DSM-5 criterion is operationalized by one item, except for one criterion (i.e., “jeopardy or losing a significant relationship, job, or educational or career opportunity because of participation in videogames”) which is represented by two items. Each criterion is rated on a 3-point Likert scale with 0 = never, 1 = sometimes, and 2 = often. For the cut-off score, a value of 0 is assigned to a criterion when the response is 'never' or 'sometimes', and a value of 1 is given when the response was 'often'. Criteria 9 and 10 are essentially the same (referring to the risk of jeopardizing significant relationships, jobs, educational or career opportunities due to videogaming), and if either one or both of these items were met, they count as one point. A final sum score is calculated, ranging from 0 to 9, with higher scores indicating that more symptoms are met. If zero criteria were met, the videogaming behavior was considered casual, whereas one or more met criteria were considered at-risk gaming behaviors in this convenience sample (Király et al., 2019).

### 2.3 Statistical analyses

Pearson’s *r* was calculated for correlation analyses. Differences between groups (casual /at-risk) were indicated by Chi2 tests, two-sample t-tests, or multivariate and univariate analyses of variance (additional differences testing for game genre, see supplementary material). Correlational analyses and difference testing were conducted with SPSS for Windows (version 28, IBM Corp., Armonk, NY, USA). Structural equation modeling was conducted using MPlus 8 (Muthén & Muthén, 2011). Model fit was evaluated with standardized root mean square residual (SRMR) and root mean square error of approximation (RMSEA), where values < 0.08 indicate a good fit with the data. Comparative fit index and Tucker-Lewis fit index (CFI/TLI) above 0.90 represent a good fit, those above 0.95 an excellent fit with the data (Hu and Bentler, 1995, Hu and Bentler, 1999). Moreover, a degrees of freedom ratio (χ2/df) < 3 is considered satisfactory (Schermelleh-Engel et al., 2003). As a requirement for mediation analyses, all manifest variables of the structural equation model correlated with each other (Baron & Kenny, 1986; Table 3). None of the variables were normally distributed as indicated by significant Shapiro-Wilk and Kolmogorov-Smirnov Tests (*p* <.01). We therefore followed the recommendation of Maydeu-Olivares (2017) and used a robust maximum likelihood estimator which is robust to non-normality (i.e., MLMV). The proposed model was analyzed on a multigroup level according to manifestations of symptom severity (i.e., casual versus at-risk) using mean structure analysis, which is often used to compare group means on the proposed constructs (Dimitrov, 2006). We used the sub-sample showing casual gaming behaviors as a reference group as indicated by “0 = casual 1 = at-risk;” in the MPlus grouping syntax line. As compared to an unconstrained (i.e., freely estimated) model, path estimates were fixed in a constrained (i.e., invariance expected) model, therefore forcing the same model across the two groups to be equal. The assumption of invariance between the constrained and unconstrained models was tested by χ2 difference testing provided in MPlus via the DIFFTEST-command which is recommended for the MLMV estimator (https://stats.oarc.ucla.edu/mplus/faq/how-can-i-compute-a-chi-squared-test-for-nested-models-with-the-mlmv-or-wlsmv-estimators-difftest/).

## 3 Results

### 3.1 Descriptive statistics and correlation analyses

Descriptive statistics of the overall sample and the subgroups are presented in Table 1 and Table 2. Groups did not differ in gender distribution. There were slight differences between groups with regard to distribution of type of occupation as well as favorite game genre in the last four weeks. Within the at-risk group more participants favored first person shooter games as compared to the casual group. On a descriptive level all other genre were similarly distributed across groups. The casual and at-risk groups differed in all variables included in the SEM Table 4.

**Table 1:** Descriptive statistics of nominal variables.

	Overall (N = 439)	Casual group (*n* = 255)	At-risk group (*n* = 184)	
Variables	n/% of overall sample	n/% of casual group	n/% of at-risk group	χ^2^-test
Gender				χ^2^ = 3.16, *p* =.075
male	231/52.6 %	125/49 %	106/57.6 %	
female	208/47.4 %	130/51 %	78/42.4 %	
Occupation				χ^2^ = 15.68, *p* =.016
pupil	12/2.7 %	5/2.0 %	7/3.8 %	
student	52/11.8 %	23/9.0 %	29/15.8 %	
trainee	20/4.6 %	6/2.4 %	14/7.6 %	
employed (part-time)	65/14.8 %	44/17.3 %	21/11.4 %	
employed (full time)	244/55.6 %	150/58.8 %	94/51.1 %	
housewife/ househusband	35/8.0 %	20/7.8 %	15/8.2 %	
pensioner	11/2.5 %	7/2.7 %	4/2.2 %	
Favorite game genre (last four weeks)				χ^2^ = 16.06, *p* =.025
Massively Multiplayer Online Role-Play (MMORPG)	17/3.9 %	7/2.7 %	10/5.4 %	
Multiplayer Online Battle Arena (MOBA)	26/5.9 %	17/6.7 %	9/4.9 %	
Action&Adventure	98/22.3 %	61/23.9 %	37/20.1 %	
First Person Shooter (FPS)	64/14.6 %	26/10.2 %	38/20.7 %	
Sport Spiele / Beat’em ups	51/11.6 %	30/11.8 %	21/11.4 %	
Simulation games	79/18.0 %	50/19.6 %	29/15.8 %	
Roleplay	33/7.5 %	16/6.3 %	17/9.2 %	
Strategy games	71/16.2 %	48/18.8 %	23/12.5 %

**Table 2:** Descriptive statistics of continuous variables.

	Overall (N = 439)		Casual group (*n* = 255)	At-risk group (*n* = 184)	
Variables	*M* (*SD*)	min–max	*M* (*SD*)	*M* (*SD*)	*t*-test
Age	34.31 (8.92)	18–49	35.49 (8.39)	32.68 (9.39)	*t*(366.81)^1^ = 3.23, *p* ≤ 0.001, *d* = 0.32^2^
Average game time a week (7 days, in h)	12.87 (19.57)	0–360	9.79 (9.09)	17.15 (27.75)	*t*(211.51)^1^ = -3.47, *p* ≤ 0.001, *d* = -0.38^3^
Average game time weekend (Saturday and Sunday, in h)	5.89 (5.21)	0–48	4.73 (4.21)	7.50 (5.99)	*t*(308.50)^1^ = -5.37, *p* ≤ 0.001, *d* = -0.55^3^
IGDT10 criteria	1.0 (1.63)	0–9	0.0 (0.00)	2.38 (1.75)	*t*(1 8 3)^1^ = -18.40, *p* ≤ 0.001, *d* = -2.10^3^
Urge: frequency per week	8.67 (48.44)	1–1000	4.22 (3.9)	14.83 (74.36)	*t*(183.73)^1^ = -1.93, *p* =.055, *d* = -0.22^3^
Urge: strength	51.98 (23.00)	1–100	44.43 (22.59)	62.45 (19.18)	*t*(425.56)^1^ = -9.01, *p* <.001, *d* = -0.85^3^
Urge: frequency conflict	37.15 (27.82)	0–100	29.06 (26.85)	48.36 (25.18)	*t*(4 3 7) = -7.63, *p* <.001, *d* = -0.74^3^
UPPS-P: negative urgency	9.57 (2.74)	4–16	9.04 (2.54)	10.29 (2.85)	*t*(4 3 7) = -4.81, *p* <.001, *d* = -0.47^3^
UPPS-P: lack in premediation	7.55 (2.31)	4–15	7.38 (2.32)	7.78 (2.28)	*t*(4 3 7) = -1.78, *p* =.075, *d* = -0.17^3^
UPPS-P: lack in perseverence	7.27 (2.33)	4–16	7.07 (2.37)	7.54 (2.25)	*t*(4 3 7) = -2.11, *p* =.036, *d* = 0.20^3^
UPPS-P: sensation seeking	9.76 (2.39)	4–16	9.36 (2.40)	10.32 (2.27)	*t*(4 3 7) = -4.23, *p* <.001, *d* = -0.41^3^
UPPS-P: positive urgency	9.78 (2.29)	4–16	9.37 (2.06)	10.34 (2.47)	*t*(349.71)^1^ = -4.34, *p* <.001, *d* = -0.43^3^

*Note***.**^1^Equal variances not assumed as indicated by significant Levene’s tests; ^2^ Two-sided *t*-test; ^3^ One-sided *t*-test.

**Table 3:** Correlation analyses of manifest study variables.

	*M* (*SD*)	1)	2)	3)	4)	5)	6)	7)	8)	9)	10)	11)	12)	13)	14)	15)	16)	17)
01) Absorption	2.36 (0.82)	1																
02) Flow	2.68 (0.73)	0.714**	1															
03) Immersion^1^	3.83 (1.07)	0.413**	0.480**	1														
04) Presence	3.34 (0.76)	0.548**	0.647**	0.459**	1													
05) Gratification of needs	1.94 (0.87)	0.489**	0.539**	0.405**	0.411**	1												
06) Experience of pleasure	2.91 (0.60)	0.357**	0.404**	0.455**	0.365**	0.536**	1											
07) Compensation of needs	1.42 (1.08)	0.471**	0.430**	0.243**	0.342**	0.574**	0.299**	1										
08) Experience of relief	1.68 (1.12)	0.440**	0.431**	0.277**	0.364**	0.521**	0.364**	0.800**	1									
09) CASBA_01 R/RC: … *would be best to improve my mood.*	2.76 (1.22)	0.260**	0.276**	0.228**	0.278**	0.392**	0.360**	0.377**	0.383**	1								
10) CASBA_02 PC: … *would cause an intense sense of well-being in myself.*	2.80 (1.19)	0.334**	0.353**	0.304**	0.356**	0.454**	0.470**	0.413**	0.433**	0.740**	1							
11) CASBA_03 OC: … *is the most urgent thing that I’d like to do immediately.*	1.38 (1.33)	0.431**	0.452**	0.269**	0.316**	0.488**	0.287**	0.457**	0.388**	0.551**	0.507**	1						
12) CASBA_04 R/RC: … *would give me a sense of satisfaction.*	2.41 (1.30)	0.446**	0.481**	0.324**	0.402**	0.425**	0.515**	0.355**	0.389**	0.489**	0.580**	0.539**	1					
13) CASBA_05 OC: … *is something I wish so much that my heart beats faster.*	1.07 (1.37)	0.482**	0.470**	0.247**	0.334**	0.477**	0.247**	0.424**	0.345**	0.442**	0.428**	0.712**	0.529**	1				
14) CASBA_06 R/RC: … *would be the most effective way of rewarding myself.*	1.95 (1.47)	0.422**	0.444**	0.323**	0.370**	0.440**	0.341**	0.447**	0.466**	0.557**	0.579**	0.572**	0.498**	0.630**	1			
15) CASBA_07 PC: … *would make me feel better physically.*	1.69 (1.46)	0.424**	0.447**	0.247**	0.339**	0.490**	0.303**	0.474**	0.465**	0.548**	0.521**	0.629**	0.433**	0.622**	0.649**	1		
16) CASBA_08 R/RC: … *would make me less stressed.*	2.72 (1.36)	0.303**	0.305**	0.222**	0.271**	0.256**	0.288**	0.353**	0.430**	0.538**	0.490**	0.375**	0.417**	0.372**	0.491**	0.526**	1	
17) CASBA_09 OC: … *is something I miss so much that I can hardly resist it.*	0.85 (1.27)	0.417**	0.416**	0.197**	0.277**	0.439**	0.197**	0.443**	0.334**	0.389**	0.381**	0.658**	0.464**	0.752**	0.560**	0.588**	0.356**	1

*Note*. **p <.001; ^1^Subscale consists of one item. English item translations were taken from the supplementary material of Antons et al. (2019).

OC: Obsessive craving; PC: Physiological craving; RRC: Reward/Relief craving,

**Table 4:** Difference testing on relevant study variables between the casual and at-risk group.

	Casual group	At-risk group	
Variables	*M* (*SD*)	*M* (*SD*)	Two-tailed *t*-test
Absorption	2.23 (0.77)	2.53 (0.86)	*t*(368.06)^1^ = -3.67, *p* ≤ 0.001, *d* = -0.362
Flow	2.49 (0.69)	2.95 (0.69)	*t*(4 3 7) = -6.88, *p* ≤ 0.001, *d* = -0.666
Immersion^1^	3.68 (1.08)	4.04 (1.03)	*t*(4 3 7) = -3.52, *p* ≤ 0.001, *d* = -0.341
Presence	3.14 (0.71)	3.61 (0.75)	*t*(4 3 7) = -6.67, *p* ≤ 0.001, *d* = -0.645
Gratification of needs	1.76 (0.80)	2.19 (0.91)	*t*(362.39)^1^ = -5.04, *p* ≤ 0.001, *d* = -0.500
Experience of pleasure	2.83 (0.55)	3.01 (0.65)	*t*(352.35)^1^ = -3.07, *p* ≤ 0.001, *d* = -0.305
Compensation of needs	1.18 (1.00)	1.76 (1.09)	*t*(4 3 7) = -5.72, *p* ≤ 0.001, *d* = -0.553
Experience of relief	1.44 (1.08)	2.02 (1.08)	*t*(4 3 7) = -5.58, *p* ≤ 0.001, *d* = -0.539
Craving	15.08 (7.99)	21.15 (9.59)	*t*(351.50)^1^ = -7.05, *p* ≤ 0.001, *d* = -0.701

*Note***.***n* (casual group) = 255, *n* (at-risk group) = 184; ^1^Equal variances not assumed as indicated by significant Levene’s tests.

## 4 Group comparisons

### 4.1 Structural equation model

According to Wegmann et al. (2022), the experience of gratification and compensation are reinforcement mechanisms that both contribute to reward-based learning wherefore their source of variance might be similar. Thus, we allowed the covariance of both factors within the model. The final model for the multi-sample showed an acceptable fit with the data. The CFI was 0.890, TLI was 0.869, RMSEA was 0.075 (*p* <.001), and the SRMR was 0.071. χ2(2 3 0) is 514.881 (*p* <.001) with χ2/df being 2.23. The constrained and unconstrained model significantly differed from one another (χ2(12) = 22.241, *p* =.035), indicating that estimated pathways were not equal among the casual and at-risk groups.

**Fig. 1:**
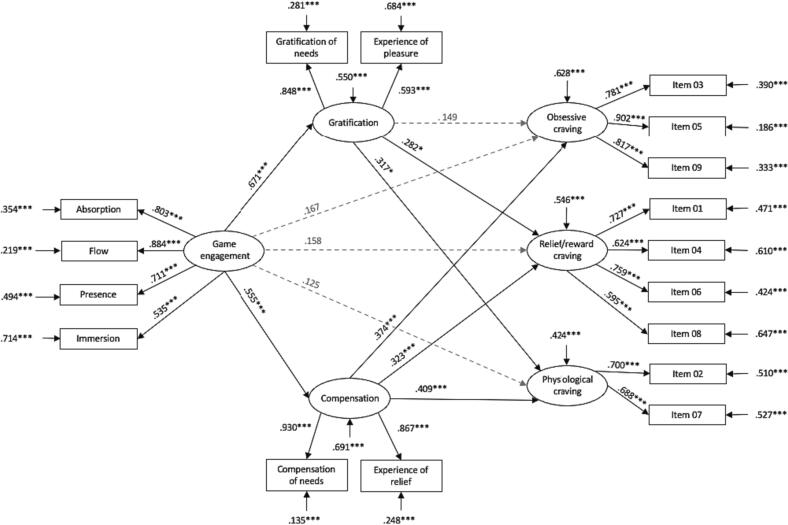
*Results of the structural equation model in the casual group.****Note****.* ***p≤ 0.001, **p≤ 0.01, *p≤ 0.05; *n* = 255.

In the casual group, the explained variance within obsessive craving was 37.2 % (*R*^2^ = 0.372, *SE* = 0.057, *p* <.001), 45.4 % within reward/relief craving (*R*^2^ = 0.454, *SE* = 0.068, *p* <.001), and 57.6 % within physiological craving (*R*^2^ = 0.576, *SE* = 0.081, *p* <.001). Overall, 45 % (*R*^2^ = 0.450, *SE* = 0.073, *p* <.001) of gratification variance and 30.9 % (*R*^2^ = 0.390, *SE* = 0.060, *p* <.001) of compensation variance were explained by game engagement. Direct pathways through the model are depicted in Fig. 1. Indirect pathways through the model are listed in Table 5.

**Table 5:** Indirect effects through the hypothesized model for the casual group.

Effects from Gaming engagement to Obsessive craving	β	*SE*	*p*
*Indirect 1* Gaming engagement → Gratification → Obsessive craving	0.100	0.079	0.207
*Indirect 2* Gaming engagement → Compensation → Obsessive craving	0.208	0.050	≤0.001
Effects from Gaming engagement to Reward/Relief craving	β	*SE*	*p*
*Indirect 1* Gaming engagement → Gratification → Reward/Relief craving	0.189	0.089	0.034
*Indirect 2* Gaming engagement → Compensation → Reward/Relief craving	0.179	0.053	≤0.001
Effects from Gaming engagement to Physiological craving	β	*SE*	*p*
*Indirect 1* Gaming engagement → Gratification → Physiological craving	0.213	0.090	0.018
*Indirect 2* Gaming engagement → Compensation → Physiological craving	0.227	0.057	≤0.001

*Note*. *n* = 255.

**Fig. 2:**
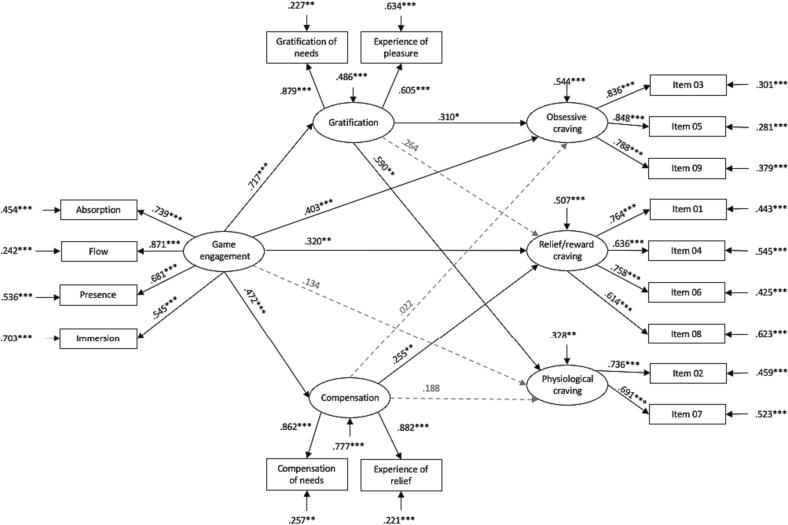
*Results of the structural equation model in the at-risk group***, *Note****.* ***p≤ 0.001, **p≤ 0.01, *p≤ 0.05; *n* = 184.

In the at-risk group, the explained variance within obsessive craving was 45.6 % (*R*^2^ = 0.456, *SE* = 0.065, *p* <.001), 49.3 % within reward/relief craving (*R*^2^ = 0.493, *SE* = 0.076, *p* <.001), and 67.2 % within physiological craving (*R*^2^ = 0.672, *SE* = 0.084, *p* <.001). Overall, 51.4 % (*R*^2^ = 0.514, *SE* = 0.101, *p* <.001) of gratification variance and 22.3 % (*R*^2^ = 0.223, *SE* = 0.062, *p* <.001) of compensation variance were explained by game engagement. Direct pathways through the model are depicted in Fig. 2. Indirect pathways through the model are listed in Table 6.

**Table 6:** Indirect *effects through the hypothesized model for the at-risk group.*

Effects from Gaming engagement to Obsessive craving	β	*SE*	*p*
*Indirect 1* Gaming engagement → Gratification → Obsessive craving	0.223	0.109	0.040
*Indirect 2* Gaming engagement → Compensation → Obsessive craving	0.010	0.053	0.844
Effects from Gaming engagement to Reward/Relief craving	β	*SE*	*p*
*Indirect 1* Gaming engagement → Gratification → Reward/Relief craving	0.176	0.114	0.123
*Indirect 2* Gaming engagement → Compensation → Reward/Relief craving	0.120	0.054	0.025
Effects from Gaming engagement to Physiological craving	β	*SE*	*p*
*Indirect 1* Gaming engagement → Gratification → Physiological craving	0.423	0.132	≤0.001
*Indirect 2* Gaming engagement → Compensation → Physiological craving	0.089	0.053	0.095

*Note*. *n* = 184.

## 5 Discussion

The current results indicate that game engagement (i.e., absorption, flow, presence, and immersion) and the experience of gratification and compensation together contribute to the experience of reward/relief, physiological, and obsessive craving in both casual and at-risk gaming groups. Considering group differences, all study variables were expressed significantly higher in the at-risk compared to the casual gaming group. In the structural equation model, all three facets of craving, reward/relief craving, physiological craving, and obsessive craving can be explained by game engagement and the experience of gratification and compensation. Descriptively, variance of facets of craving explained by game engagement and gratification/compensation was higher in the at-risk group compared to the casual gaming group. While effects of game engagement on facets of craving were almost completely mediated by gratification and compensation in the casual group (that is, except for the mediating effect of gratification in the relationship between gaming engagement and obsessive craving), only three significant mediation effects were found in the at-risk group. Overall the present results are consistent with the I-PACE model on the development and maintenance of behavioral addictions, stating that the engagement in specific behaviors may result in the experience of gratification and compensation, whose reinforcing effects increase the risk to develop craving as a central mechanism in the development and maintenance of behavioral addictions (Brand et al., 2019).

The current results indicate that game engagement may contribute to conditioning processes that are involved in the development of craving for gaming (which has been shown by results indicating higher cue-reactivity reactions in an at-risk compared to casual gaming group, Diers et al., 2023). Accordingly, games that evoke a high engagement by high levels of immersion, flow, presence, or psychological absorption may have a higher addictive potential. Previous studies have shown that especially the experience of punishment (e.g., lose a life, restart a level) and type of presentation (e.g., audio and graphics) increase the level of flow experience during gaming (Laffan et al., 2016). In addition, also games that require the player to communicate with others, to be attentional vigilant, or to react fast may have a higher addictive potential since they contribute to a feeling of immersion and flow (Brandtner et al., 2022). The results indicate that especially games inducing a high level of engagement may contribute to intense cravings by eliciting the experience of gratification and compensation, especially in casual gamers. In later stages of addictive behaviors, game engagement might be more directly involved in the development of craving. Accordingly, the level of game engagement may be one factor that can be used to identify videogames with a high addictive potential. As indicated by additional analyses (see supplementary material) one game genre that might have a higher addictive potential might be First Person Shooter games. Individuals who favored this game genre in the last four weeks show higher levels in game engagement with regard to flow and presence, higher levels of pleasure experience but did not differ with regard to craving experience. In addition, a higher proportion of individuals at-risk of developing a GD favored First Person Shooter games as compared to other game genres. However, further investigations are warranted to understand how different game genre and game characteristics contribute to the development GD. Game engagement including flow and presence are features that may be relevant.

The partial mediations of game engagement on facets of craving in the at-risk group (in contrast to almost full mediations in the casual gaming group) can be explained with an increased habituation and tolerance. Although individuals engage deeply in the game, they might not experience the same amount of gratification and compensation as individuals with a casual gaming behavior. For example, individuals with GD may be motivated by other factors such as the need for completion (King et al., 2018). This may be one mechanism resulting in the shift from reward-driven behavior to compulsive gaming in later stages of GD (Brand, 2022). However, in this at-risk group, mediating effects over gratification and compensation are still partially present, indicating that craving may be a conscious representation of anticipated reinforcement effects and may therefore still provoke goal-directed behaviors.

Interestingly, gratification and not compensation was associated with obsessive craving in the at-risk group and compensation but not gratification in the casual gaming group. Based on addiction theories (e.g., Koob, 2013), we would have expected that compensation would be more strongly associated with obsessive craving in the at-risk group. Since this is the first study that focused on effects of gratification and compensation on different facets of craving, these results need to be validated in future studies.

Gratification and compensation have strong associations with physiological craving, especially in the model with casual gamers. Previous studies have shown that mental simulations of experiences have a strong corporal influence (Andrade et al., 2012). Similarly, mental representation of experienced gratification and compensation might transit into bodily experienced craving responses. Further, although speculative, it is possible that physiological craving especially occurs when individuals experience a higher deficit when not gaming in contrast to the experience of gratification and compensation during gaming (Brandtner et al., 2021). How a deficit in gratification and compensation affects different facets of craving needs to be investigated in future studies.

## 6 Limitations

Some limitations of the current study should be considered. First, the current study was cross-sectional. Future studies should investigate with longitudinal designs, to which degree game engagement contributes to the development of craving and GD symptoms. Second, the sequence of questionnaires within the online-survey may have affected results. For example, thinking about game engagement and gratifying/compensating effects could have elicited desire thinking and thereby the current degree of craving experience (Brandtner et al., 2021). Differences between groups may be further explained by other variables such as time and frequency engaged in gaming, degree of impulsivity, or differences in game genre preferred. Systematic investigations with regard to these variables are warranted.

## 7 Conclusion

This is one of the first studies focusing on factors that contribute to the development of craving for gaming. Game engagement has been identified as a potential feature of videogames that may contribute to conditioning processes related to the experience of gratification and compensation and involved in the development of craving. While the level of game engagement may be one factor that could be used by regulatory bodies to rate the addictive potential of videogames, it should still be considered that individual factors such as the level of experienced gratification and compensation are the central mechanisms leading to the conditioning effects and not the videogame-features per se. In addition, further factors besides reinforcement learning mechanisms such as intrusive thoughts and desire thinking should be considered as relevant mechanisms resulting in craving.

## Declaration of competing interest

The authors declare that they have no known competing financial interests or personal relationships that could have appeared to influence the work reported in this paper.

## Data Availability

Data will be made available on request.
