# Supplementary material for: From game engagement to craving responses – The role of gratification and compensation experiences during video-gaming in casual and at-risk gamers
Source: Addict Behav Rep. 2023 Nov 30;18:100520. doi: 10.1016/j.abrep.2023.100520 (PMC10749870; doi:10.1016/j.abrep.2023.100520)
Supplement: Supplementary Data 1 [file mmc1.docx]

**Supplementary Material**

**S1. Multivariate (MANOVA) and Univariate Analyses of Variance (ANOVA) with favourite game genre within the last four weeks as group variable.**

|  | ***F*(7, 431)** | ***p*** | **partial eta²** |
| --- | --- | --- | --- |
| Absorption | 1.68 | .111 | .027 |
| Flow | 3.99 | **<.001** | .061 |
| Immersion | 1.75 | .095 | .028 |
| Presence | 2.42 | **.019** | .038 |
| Gratification of needs | 2.59 | **.013** | .040 |
| Experience of pleasure | 2.08 | **.045** | .033 |
| Compensation of needs | 1.03 | .413 | .016 |
| Experience of relief | 1.43 | .193 | .023 |
| Craving | 2.18 | **.035** | .034 |
| IGDT10 criteria | 2.87 | **.006** | .044 |

**Note.** MANOVA Wilks Lambda: *F*(10, 70)=1.43, *p* = .012, partial eta² = .33

**S2. Single comparisons for ANOVA.**

*Figure S2.1. Single comparisons for GEQ variables flow and presence.*

**
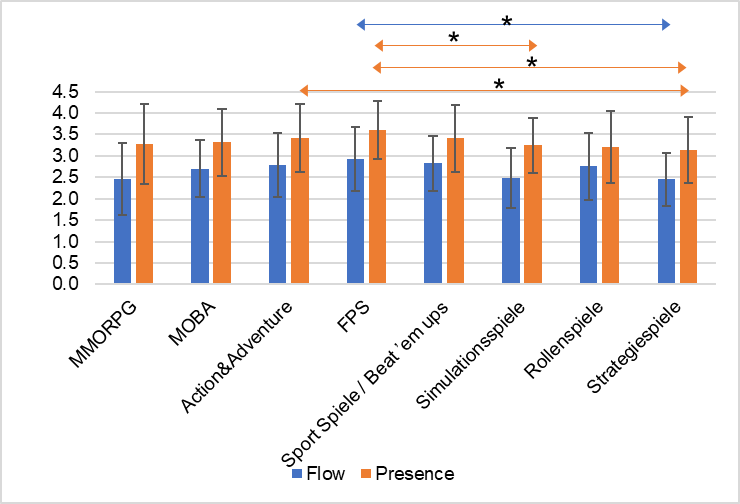
**

*Note.* MMORPG = Massively Multiplayer Online Role-Play; MOBA = Multiplayer Online Battle Arena, FPS = First Person Shooter. * *p* <.05

*Figure S2.2. Single comparisons for gratification of needs and experience of pleasure.*

**
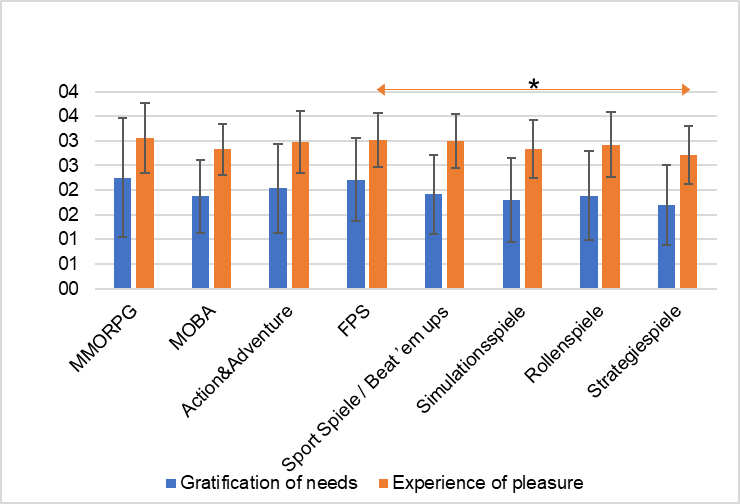
**

*Note.* MMORPG = Massively Multiplayer Online Role-Play; MOBA = Multiplayer Online Battle Arena, FPS = First Person Shooter. * *p* <.05

*Figure S2.3. Single comparisons for CASBA.*

**
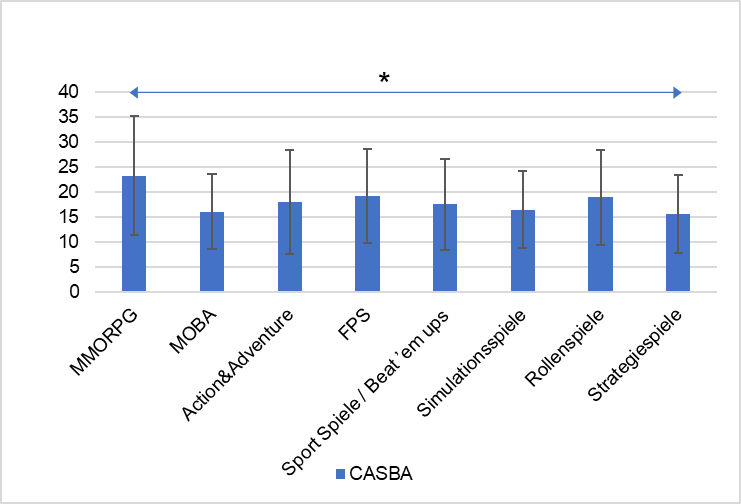
**

*Note.* MMORPG = Massively Multiplayer Online Role-Play; MOBA = Multiplayer Online Battle Arena, FPS = First Person Shooter. * *p* <.05

*Figure S2.4. Single comparisons for IGDT10 criteria.*

**
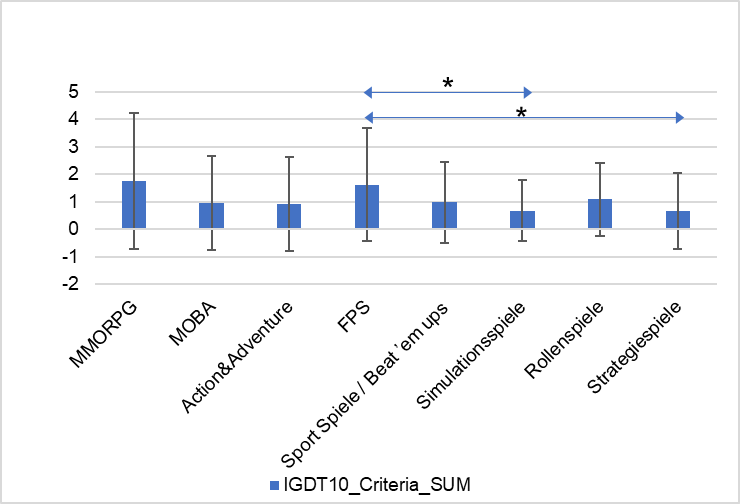
**

*Note.* MMORPG = Massively Multiplayer Online Role-Play; MOBA = Multiplayer Online Battle Arena, FPS = First Person Shooter. * *p* <.05
